# Dirofilariasis presenting as pleural effusion: a rare case report with unusual manifestations and treatment modalities

**DOI:** 10.1186/s12890-024-03154-y

**Published:** 2024-07-15

**Authors:** Rolanda Valčiukaitė-Žilinskienė, Birutė Zablockienė, Rolandas Zablockis

**Affiliations:** 1 Faculty of Medicine, Vilnius University, M.K. Ciurlionio 21, Vilnius, LT-03101 Lithuania; 2 Clinic of Infectious Diseases and Dermatovenerology, Institute of Clinical Medicine, Faculty of Medicine, Vilnius University, M.K. Ciurlionio 21, Vilnius, LT-03101 Lithuania; 3 Clinic of Chest Diseases, Immunology and Allergology, Institute of Clinical Medicine, Faculty of Medicine, Vilnius University, M.K. Ciurlionio 21, Vilnius, LT-03101 Lithuania

**Keywords:** Human, Dirofilariasis, Pleural effusion, Case report

## Abstract

**Background:**

We present an extremely rare manifestation of dirofilariasis in the pleural cavity. This is the first human pulmonary dirofilariasis reported in Lithuania; according to our knowledge, only two other patients were documented with this pathology in the world.

**Case presentation:**

A 72-year-old woman was admitted to the hospital complaining of dyspnea, left-side chest pain, and a dry cough. She was a retiree living alone in the countryside without domestic pets (sometimes stray dogs appear) or a travel history. A complete blood count was within normal limits, with a CRP level of 16.8 mg/l and D-dimer concentration of 900 µg/l, which raised suspicion of pulmonary embolism. In chest computed tomography angiography, pulmonary embolism was excluded, and only left pleural effusion without abnormal lesions was confirmed. Left thoracocentesis was performed, and the pleural fluid was evaluated as an exudate with a predominance of eosinophils (59%), along with the presence of parasites. These parasites exhibited the morphology of *Dirofilaria repens*. Oral doxycycline (100 mg, twice daily) and albendazole (400 mg, twice daily) were prescribed for a 14-day course. A month later, there were no pathological findings on the chest X-ray, and the patient no longer had respiratory symptoms. However, the patient presented with an emerged, painful palpable right breastmass, where the rash was previously observed. Ultrasound imaging revealed a 1.5 × 2 cm nodule, which was surgically removed. Parasites consistent with *Dirofilaria repens* were suspected but not definitively identified. Pharmacological treatment for dirofilariasis was not further prescribed.

**Conclusions:**

This case encourages doctors to be more vigilant because the patient, who neither travelled nor kept any pets, contracted dirofilariasis. Diagnostic and treatment guidelines are lacking, necessitating further research. Treatment with doxycycline and albendazole yielded positive outcomes, suggesting potential efficacy for dirofilarial pleuritis.

## Background

Dirofilariasis is a mosquito-transmitted infection, commonly caused by filarial nematodes *D. repens* and *D. immitis*. [1,2] The most common clinical variants of dirofilariasis are subcutaneous, ocular and pulmonary forms [3]. The main hosts are domesticated and wild dogs, carnivores, less commonly cats [4]. The nematode has five life-cycle stages (L1-L5) and completes L3 cycle in dogs or carnivores, acting as reservoirs for disease transmission to humans [2,4,5]. During a blood meal, an infected mosquito transmits larvae through the bite site into the human bloodstream. The larvae develop, and the adult migrates to the right heart ventricle, where they eventually perish. Fragments of the deceased dirofilaria enter the pulmonary arteries, causing pulmonary embolism and local inflammation [2,5–7]. Although pulmonary dirofilariasis is typically asymptomatic, in rare cases, it may manifest with symptoms such as fever, dyspnea, cough and wheezing [7]. Radiologically, small subpleural nodules, approximately 0.5–4.5 cm in size are commonly observed, which are referred to as ‘coined lesions’ [7,8]. Pleural effusion exists in very rare cases [9,10].

The escalating effects of climate warming, global movement, improved awareness of the infection contribute to the growing morbidity of dirofilariasis in humans, underscoring its increasing relevance in contemporary medicine [11,12]. In this report we present an exceptionally rare case of pleural effusion due to dirofilariasis. This case marks the first instance of human pulmonary dirofilariasis reported in Lithuania.

## Clinical case

**Fig. 1:**
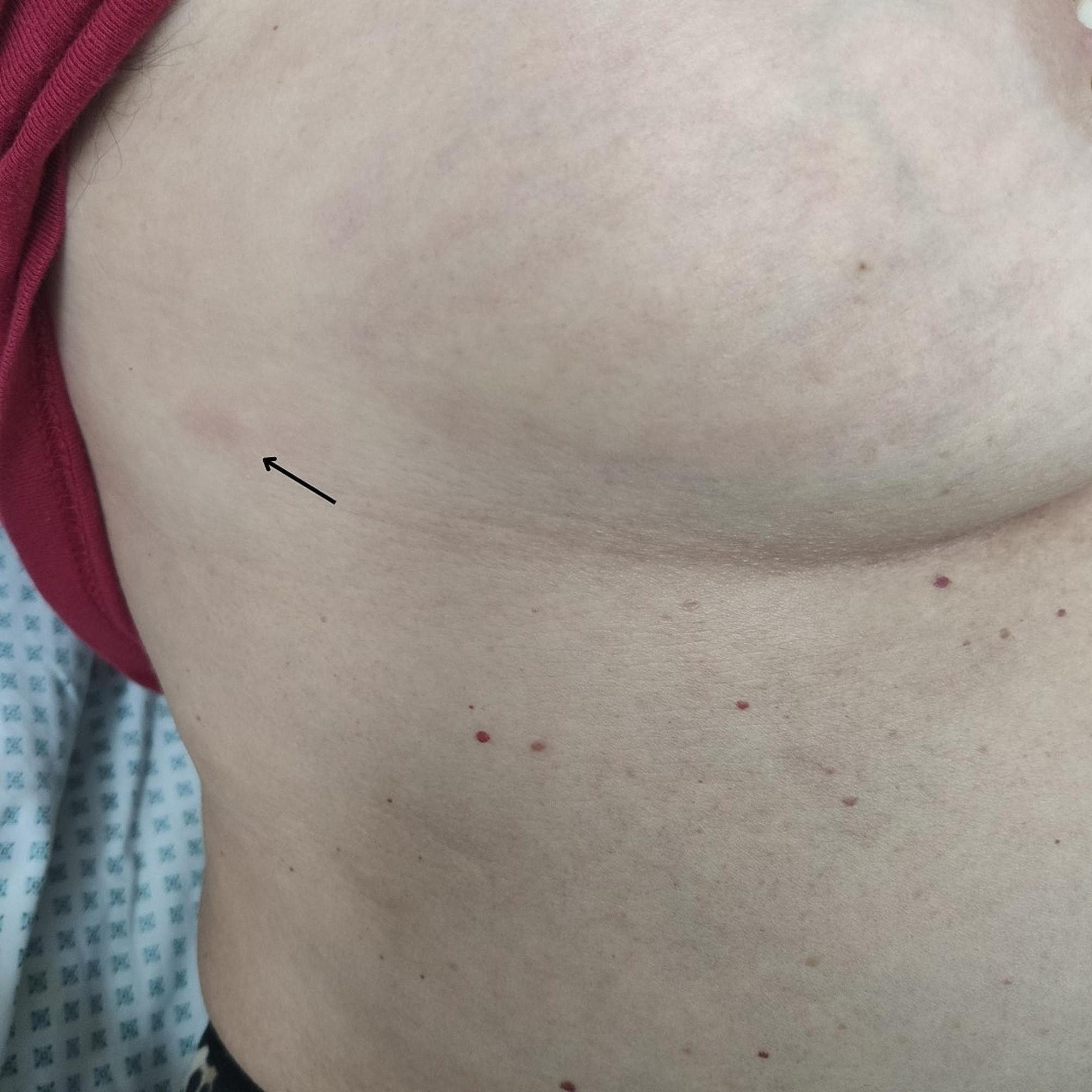
Erythematous skin lesion on the right axillary region

A 72-year-old-woman was admitted to Vilnius University Hospital Santaros Klinikos with complaints of shortness of breath, left-side chest pain and a dry cough. A retiree living alone in the countryside without domestic pets (sometimes stray dogs appear) or a travel history, she had no previous oncological disorders. Upon physical examination, her SpO2 level was 96% at rest, with a respiratory rate of 18 breaths per minute. No vesicular wheezing was detected on the left lung below the scapular margin. Notably, a clinical examination revealed erythematous rashes on the right breast and left forearm (Fig. 1).

**Fig. 2:**
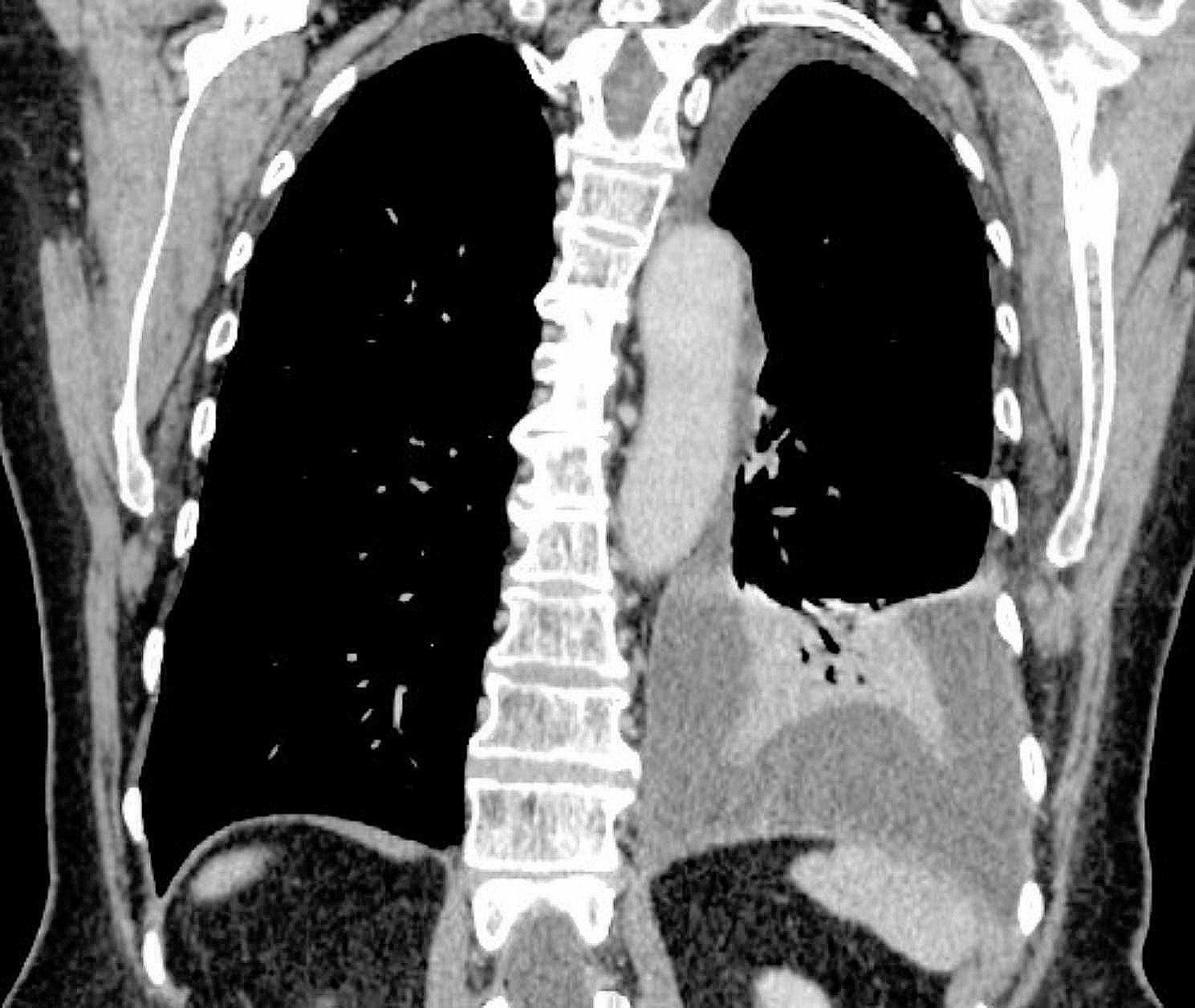
CT scan of pleural fluid

A complete blood count was within normal limits, but a slightly elevated CRP level of 16.8 mg/l and D-dimer concentration of 900 µg/l raised suspicion of pulmonary embolism. In chest computed tomography angiography pulmonary embolism was excluded, only left pleural effusion without abnormal lesions was confirmed (Fig. 2).

**Fig. 3:**
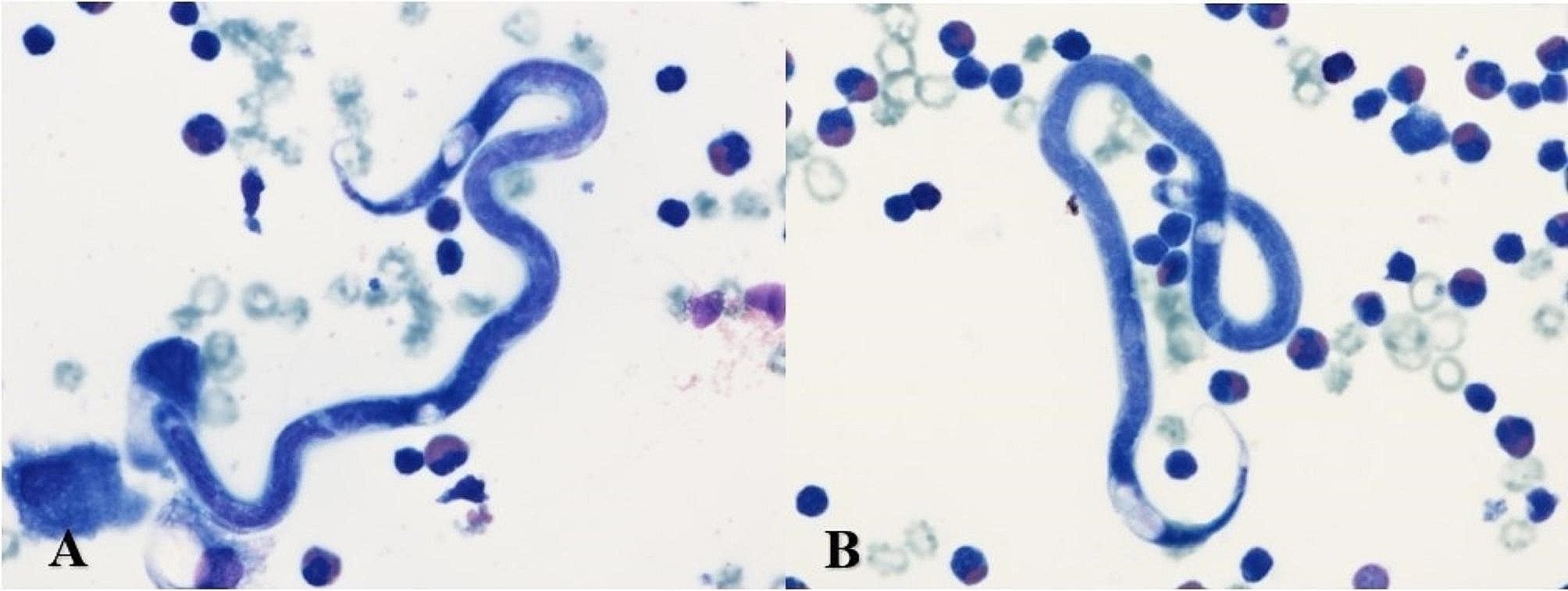
Microfilaria of *Dirofilaria repens* from the pleural fluid. Image author: MD V. Dumbrauskienė

The patient was hospitalized in the Pulmonology and Allergology department, where diagnostic thoracocentesis revealed a predominance of eosinophils (59%) in the exudative pleural effusion, along with the presence of parasites. These parasites exhibited the morphology of *Dirofilaria repens* (Fig. 3).

The patient received a diagnosis of dirofilariasis. Dirofilariasis usually involves subcutaneous tissue, therefore a left forearm‘s skin lesion was biopsied, but there were no specific findings. The patient was consulted by an infectious disease specialist and prescribed oral doxycycline (100 mg, twice daily) and albendazole (400 mg, twice daily) for a 14-day course. A day after routine tests, she was diagnosed with COVID-19, exhibiting no respiratory insufficiency. Her health remained stable, and she was discharged earlier with a continued course of doxycycline and albendazole. The patient completed the full medication course and underwent outpatient monitoring by a pulmonologist and an infectious disease specialist.

A month after administering treatment, a chest x-ray revealed no pathological findings and the pleural effusion had resolved. However, the patient presented with an emerged, painful, palpable right breast mass in the same location where the rash was observed. Ultrasound imaging revealed a 1,5 × 2 cm nodule, surgically removed, revealing parasites that were consistent with *Dirofilaria repens* but not definitively identified at that time. Pharmacological treatment for dirofilariasis was not further prescribed. Subsequent follow-up visits indicated no recurrence of new subcutaneous and pulmonary nodules or pleural effusion, maintaining the patient’s overall health stability.

## Discussion and conclusions

This case report is noteworthy for several reasons. Firstly, it marks the initial clinical instance of dirofilariasis presenting with pleural effusion in Lithuania.

**Fig. 4:**
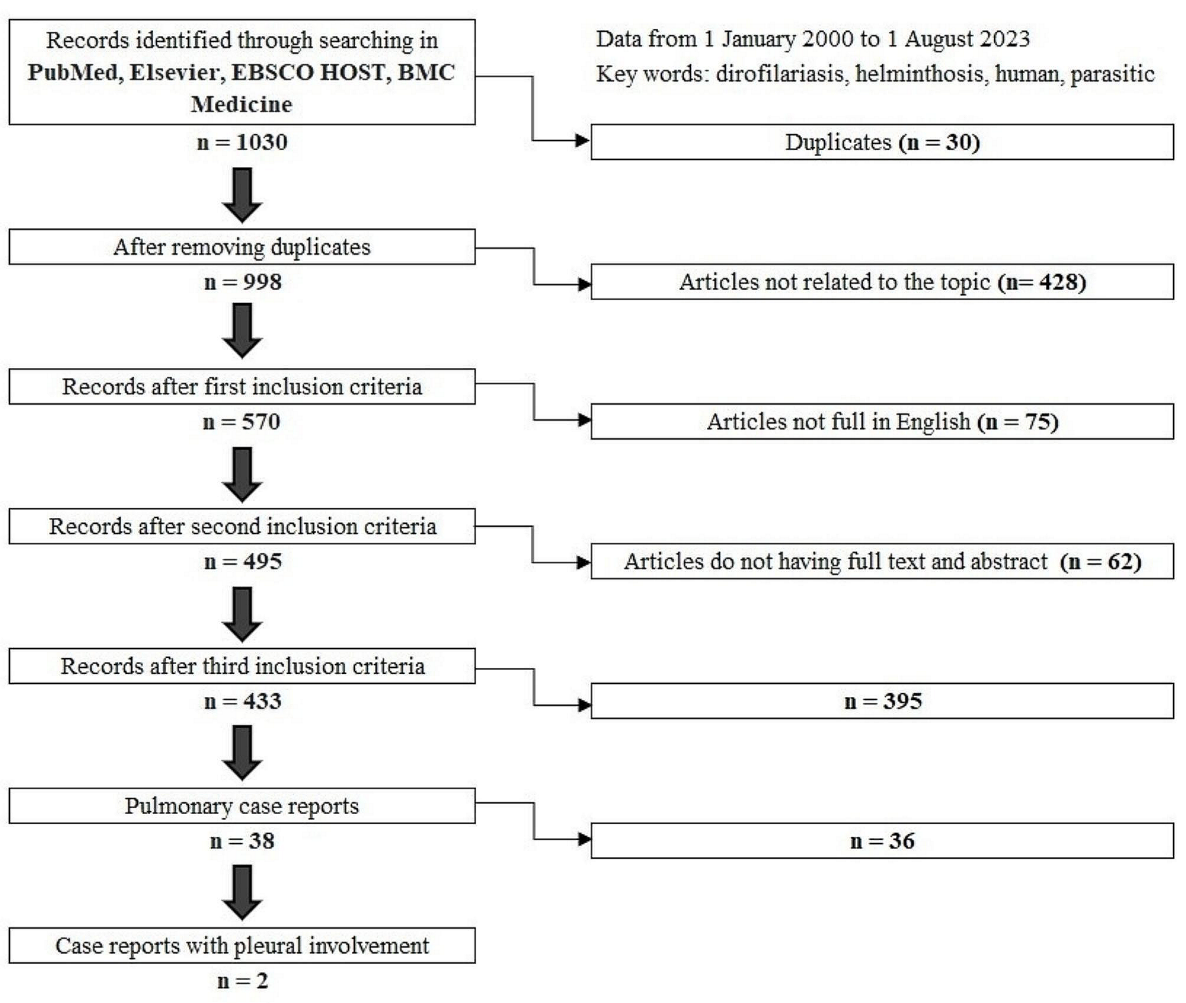
Detailed scheme of literature search and exclusion criteria

Secondly, dirofilariasis causing pleural effusion is exceptionally rare. Our comprehensive literature search, spanning databases such as PubMed, Elsevier, EBSCO HOST, and BMC Medicine from January 1, 2000, to August 1, 2023, using MeSH headings “human”, “dirofilariasis”, “helminthosis” and “parasitic”, identified scant references discussing pleuritis caused by dirofilariasis. The inclusion criteria comprised articles within the scope of our focus, fully written in English, and possessing both full text and an abstract. The detailed literature search and selection scheme is presented in Fig. 4. Remarkably, only two clinical cases of dirofilariasis pleurisy meeting the inclusion criteria were discovered (Table 1) [9,10]. During the search, clinical cases caused by other parasites within the filariasis family, such as *Wuchereria bancrofti*, were identified. However, these cases were not included as they did not pertain to the genus *Dirofilaria* [13–17].

**Table 1:** Cases of dirofilariasis with pleural involvement

	Naoyuki Yoshino et al., 2003 [9]	Helena Biasizzo et al., 2022 [8]
Country	Japan	Slovenia
Gender	Female	Female
Age	53 years	40 years
Travel history	N/I	Yes, 2020 Croatia
Dog owner	N/I	No
Symptoms	Cough	Skin lesions in the upper trunk and axillary regions; Dyspnoea, dry cough, pain in the left hemithorax, night sweats, general malaise
Localisation	Pulmonary, pleural	Cutaneous, pulmonary, pleural
Radiological findings	Nodular mass in the lower right lung, left pleural effusion.	Left-sided pleural effusion, lesion adjacent to the left posterobasal pleura, pleural thickening, reactive mediastinal lymph nodes
Pleural fluid characteristics	N/I^†^	Exudative pleurisy with lymphocytic predominance
Histology	*D. immitis* from the resected tumour	*D. repens* in pleural fluid
Treatment	Lower right lobectomy	Ivermectin 200 µg/kg/d for 4 days Doxycycline mg 2 x/d for 7 days

† N/I – no information

All three clinical cases of dirofilariasis, including the one under consideration, involved women, aligning with scoping review analyses indicating a higher prevalence of dirofilariasis in women, particularly those aged 50–59 years [3].

Scientific research underscores that the majority of dirofilariasis cases are concentrated in southern Europe, particularly Italy [11,18]. Not only traveling emerge as a significant risk factor for heartworm disease, but so does the movement of infected animals [18]. While the patient in our presented clinical case had not recently travelled abroad and had no domestic pets, it is crucial to note that dirofilariasis cases are on the rise in endemic countries, with the Baltic countries being no exception [19,20].

Our clinical case stands out for the occurrence of dirofilariasis in two distinct localizations. Typically, after a mosquito bite, the infected larva perishes without inducing symptoms. In rarer cases, it develops, causing local reactions such as erythema, redness and itching [21]. Even more infrequently, microfilariae enter the bloodstream and migrate to other organ systems, as observed in our clinical case. Despite the fact that dirofilariasis was not detected in the biopsy of left forearm, no biopsy of the right breast rash was performed, so we can assume that it could have been the primary source of infection. This speculation is supported by the subsequent discovery of worms upon surgical excision of the mass, although their specific identification remains elusive.

Pleural punctate cytology characteristic of dirofilariasis lacks available data. Given its parasitic origin, it can be assumed that the punctate should correspond to a pleural parasitic infestation, characterized by an exudate according to Light’s criteria, with a predominance of eosinophils ≥ 10% in the pleural fluid [22,23]. Such characteristics were evident in our presented case report. In a clinical case from Slovenia, the pleural fluid displayed a predominance of lymphocytes [9]. In the literature, it is recommended to rule out tuberculosis when a punctate of lymphocytic origin is detected, which was done in the clinical case from Slovenia [24].

Pulmonary dirofilariasis is a self-limiting disease and in the absence of symptoms, specific treatment is unnecessary [7,21]. Surgical excision of the nodule is the preferred treatment when symptoms arise [7,21,25,26]. Medical treatment is advised when total excision of the nodule or surgical intervention is impractical, or the patient is immunocompromised [21]. Due to the rarity of dirofilariasis, established medical treatment recommendations are lacking. Individual research studies suggest that medical treatment with doxycycline, coupled with albendazole or ivermectin, inhibits microfilarial migration and induces a long-term reduction [21,27].

Our clinical case is unique due to manifestations in different localizations and both surgical and medical treatment options used. In our clinical report, where no primary nodule was identified and pleural fluid predominated, surgical treatment was not initially possible. Based on the reviewed studies, treatment with doxycycline and albendazole was prescribed and proved effective for dirofilarial pleuritis, as the patient’s respiratory symptoms have disappeared. Only later, when a subcutaneous focus was discovered, the surgical treatment was applied.

This patient represents a singular case of pleural effusion caused by dirofilariasis, an extraordinarily rare pathology documented in only two other patients, according to literature data. This case is unique as the individual, who neither travelled nor kept any pets, contracted dirofilariasis. Consequently, it can be inferred that future rises in travel, animal mobility, and global warming may contribute to an increased incidence of dirofilariasis cases. This escalation may affect not only travellers but also individuals leading less-travelled lifestyles.

Owing to the scarcity of instances, diagnostic and treatment guidelines for this condition are lacking. However, our clinical case suggests that conservative treatment with doxycycline and albendazole yielded positive outcomes for dirofilarial pleuritis.

## Data Availability

The data underlying this case report will not be shared publicly to respect the privacy of the patient described here. All data generated or analysed during this study are included in this published article [and its supplementary information files].
